# Fungi stabilize connectivity in the lung and skin microbial ecosystems

**DOI:** 10.1186/s40168-017-0393-0

**Published:** 2018-01-15

**Authors:** Laura Tipton, Christian L. Müller, Zachary D. Kurtz, Laurence Huang, Eric Kleerup, Alison Morris, Richard Bonneau, Elodie Ghedin

**Affiliations:** 1 Department of Computational and Systems Biology, University of Pittsburgh School of Medicine, Pittsburgh, 15260 PA USA; 2 Center for Genomics and Systems Biology, New York University, New York, 10003 NY USA; 3 Flatiron Institute, Center for Computational Biology, Simons Foundation, New York, 10010 NY USA; 4 Department of Microbiology, New York University School of Medicine, New York, 10016 NY USA; 5 Division of Pulmonary and Critical Care Medicine and HIV/AIDS Division, University of California San Francisco, San Francisco, 94110 CA USA; 6 Division of Pulmonary and Critical Care, Department of Medicine, David Geffen School of Medicine, University of California Los Angeles, Los Angeles, 90095 CA USA; 7 Division of Pulmonary, Allergy and Critical Care Medicine, University of Pittsburgh School of Medicine, Pittsburgh, PA USA; 8 Department of Epidemiology, College of Global Public Health, New York University, New York, NY USA

## Abstract

**Background:**

No microbe exists in isolation, and few live in environments with only members of their own kingdom or domain. As microbiome studies become increasingly more interested in the interactions between microbes than in cataloging which microbes are present, the variety of microbes in the community should be considered. However, the majority of ecological interaction networks for microbiomes built to date have included only bacteria. Joint association inference across multiple domains of life, e.g., fungal communities (the mycobiome) and bacterial communities, has remained largely elusive.

**Results:**

Here, we present a novel extension of the SParse InversE Covariance estimation for Ecological ASsociation Inference (SPIEC-EASI) framework that allows statistical inference of cross-domain associations from targeted amplicon sequencing data. For human lung and skin micro- and mycobiomes, we show that cross-domain networks exhibit higher connectivity, increased network stability, and similar topological re-organization patterns compared to single-domain networks. We also validate in vitro a small number of cross-domain interactions predicted by the skin association network.

**Conclusions:**

For the human lung and skin micro- and mycobiomes, our findings suggest that fungi play a stabilizing role in ecological network organization. Our study suggests that computational efforts to infer association networks that include all forms of microbial life, paired with large-scale culture-based association validation experiments, will help formulate concrete hypotheses about the underlying biological mechanisms of species interactions and, ultimately, help understand microbial communities as a whole.

## Background

Determining networks of microbial interactions that affect the fitness of individual species is relevant for the functional characterization of a microbial community. These interactions can vary across time and space, depending on both abiotic and biotic factors. Common abiotic factors include oxygen, temperature, and pH, while biotic factors can include the presence or absence of other microbes. The ability to predict biological associations between microbes from next-generation sequencing data, particularly from targeted amplicon sequencing (TAS), has been a topic of increasing interest due to the advent of efficient statistical network inference tools for TAS data [1–3]. The resulting microbial association networks can be informative both at species and community levels. At the species level, associations have been used to successfully co-culture organisms previously thought un-culturable. Co-culture has, for example, enabled the cultivation and sequencing of a member of the candidate division TM7, called TM7x, from the human oral microbiome [4]. TM7x is now known to be an obligate epibiont of *Actinomyces odontolyticus* and cannot be cultured without it. At the community level, the topological organization of the association network can indicate ecological and co-evolutionary relationships in the community [2]. For example, early-life antibiotic exposure has been shown to have lasting and transferable effects on the topology of murine gut microbial association networks [5].

Standard methods for microbial association inference from TAS data are based on co-occurrences or correlations [1, 3] and have been applied to bacterial communities. By targeting the 16S subunit of the ribosomal RNA gene (16S rRNA gene), these bacterial studies ignore other components of the community, including fungi. Although present at significantly lower abundance than bacteria, fungi play an important role in the microbial community, and interactions between individual fungi and bacteria are well documented [6–8], making these interactions deserving of further study [9].

Here, we present a statistical framework that allows the inference of associations across multiple microbial domains. Rather than relying on correlation or co-occurrence information, we consider a cross-domain extension of the SPIEC-EASI (SParse InversE Covariance estimation for Ecological ASsociation Inference, pronounced “speak easy”) method [2], which estimates microbial associations in a compositionally robust, sparse graphical modeling framework. The original SPIEC-EASI method relies on a normalization technique to make the operational taxonomic units (OTUs) independent of one another. We exploited this pre-processing step to expand the method to include multiple domains, as described in detail in the methods section. By using independent TAS studies of the 16S rRNA gene and Internal Transcribed Spacer (ITS) from the same samples of bacterial and fungal communities, our novel SPIEC-EASI variant allows compositionally robust, simultaneous inference of both within-domain and cross-domain associations. The inferred network represents a parsimonious statistical description of cross-domain species associations, which could result from direct species interactions or relationships mediated through latent (unmeasured) biotic or abiotic factors.

We consider two microbiome studies that include both 16S rRNA gene and ITS sequencing: the lung microbiome [10, 11], and the skin microbiome [12, 13]. For both habitats, we highlight system-wide features of the single-domain and cross-domain networks, as well as key re-organization patterns of the cross-domain association networks compared to their single-domain counterparts. For the lung microbiome, we highlight network modules of disease-associated microbes. Guided by the inferred skin microbiome association network, we select several skin microbiome community members, belonging to two bacterial and one fungal species, and experimentally confirm their predicted co-variation profiles by co-culture.

## Results

To highlight the relevance and the impact of cross-domain associations on community organization, we inferred association networks of two microbiome communities. The first community was the lung microbiome from the Pittsburgh cohort of the Lung HIV Microbiome Project [10, 11]. The cohort included both HIV-infected and HIV-uninfected individuals and these individuals had either normal lung function or chronic obstructive pulmonary disease (COPD). There were 25 individuals with a total of 35 bronchoalveolar lavage (BAL) samples. The second community was the skin microbiome from the National Human Genome Research Institute [12, 13]. This cohort consisted of 10 healthy individuals with 382 skin swab or nail clipping samples obtained from 14 body sites. We inferred three association networks for each microbiome: a single-domain bacterial (SDB), a single-domain fungal (SDF), and a cross-domain bacterial-fungal (CDBF) network. For each habitat, we analyzed the topology of the networks in terms of modularity and connectivity, the impact of including both microbial domains on the re-organization of the networks, network assortativity of species with respect to phyla, distributions of interaction strengths, and attack robustness.

### Cross-domain interactions add connectivity and reduce modularity of interaction networks

#### Lung microbiome

**Fig. 1:**
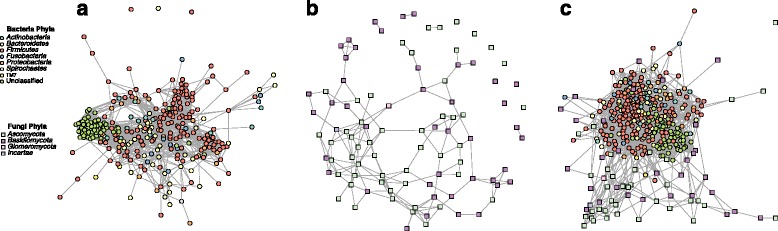
Lung microbiome networks. Networks inferred for the lung microbiome based on **a** bacteria only, **b** fungi only, and **c** bacteria and fungi combined. In all three networks, bacterial nodes are circles and fungal nodes are squares. Each node is colored by phyla. Edges between nodes represented a predicted interaction, either positive or negative

The SDB network derived from the lung microbiome dataset was comprised of one large connected component (302 out of 305 OTUs (99.01%)) and three singleton OTUs with no connections to the main network (Fig. 1a). The average number of association partners (node degree) for each OTU was 15.75 (SD: 10.70). Based on their connectivity pattern, we clustered the OTUs using modularity maximization [14], resulting in 7 modules (i.e., potential niches) with realized modularity [15] (the ratio of the number of edges within modules and the number of edges across modules) of 0.85. Following Estrada’s topological classification of complex networks [16], the SDB network belonged to class II, characterized by a highly modular organization and no central core, implying that there are several groupings of species that only require species within each group to interact. There was a high degree of assortativity of the network with respect to phylum (assortativity coefficient *c*_a_ = 0.56).

The SDF network of the lung microbiome contained one large connected component (83.33% of OTUs), four dyads (8.33%), eight singletons (8.33%), and an average node degree of 3.46 (SD, 2.35) (Fig. 1b). The large connected component was comprised of eight modules with realized modularity of 0.80. The SDF network belonged to Estrada class IV, which contains networks that can be understood as a mixture of a core-periphery and modular networks, implying the combination of a core set of species and multiple groupings. No significant assortativity with respect to phylum was observed (*c*_a_ = − 0.01).

The CDBF network of the lung microbiome consisted of one large connected component with 369 bacterial and fungal nodes and one singleton fungal node; it showed an average node degree of 16.12 (SD, 9.78) (Fig. 1c). The singleton fungal OTU was identified as *Candida dubliniesis*, which in the SDF network was connected only to the fungus *Plicaturopsis crispa*, an OTU not present in the combined cross-domain dataset. The CDBF network was comprised of eight modules with reduced realized modularity of 0.68, implying that the individual modules had more associations among each other. Like the SDB network, the lung CDBF network belonged to Estrada class II, showing modular organization without central core, indicative of interaction within groupings rather than across them. Assortativity of the CDBF network with respect to phylum (*c*_a_ = 0.45) was slightly reduced compared to the SDB network.

When we compared the CDBF network to the domain-specific networks in terms of connectivity and robustness, we found that the average path length was significantly shorter in the cross-domain network. In fact, the average (or characteristic) path length between any two bacterial nodes in the cross-domain network was 2.588 (SD, 0.734) and between any two fungal nodes was 3.605 (SD, 1.508). Each path length was reduced by more than one compared to the single-domain networks: the SDB (mean, 3.176; SD, 1.094, *t* test *p* < 0.0001) and SDF (mean, 4.549; SD, 2.203; *p* < 0.0001). We computed the total expected commute time (ECT), a global network property which can be understood as a measure of how efficiently processes (such as movement, gene flow, or metabolites) can diffuse over the entire association network [17, 18]; smaller ECTs imply more efficient *global* connectivity. We observed that the CDBF network is more efficiently connected (ECT = 1613) than the SDB network (ECT = 1876) despite its larger number of community members, suggesting that fungi facilitate improved communicability in the microbial ecosystem.

**Fig. 2:**
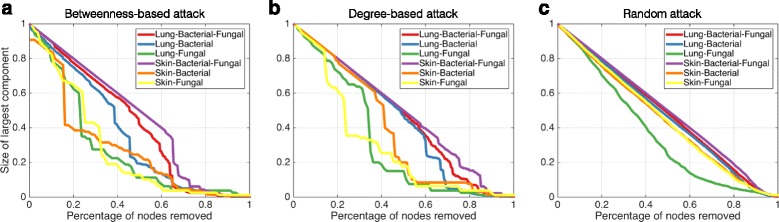
Robustness curves for all networks. Attack robustness of a network was measured by sequentially removing nodes based on the node’s **a** betweenness, **b** degree, or **c** randomly selected and measuring the percentage of nodes that remain in the central connected component. Measurement of robustness was performed for each of our six networks and the results are plotted here with the percentage of nodes removed on the *X* axis and the percentage of remaining nodes in the central connected component on the *Y* axis. Each network is represented by a line on this graph. A larger area under the curve indicates a more robust network

To quantify the influence of species loss on network connectivity, we followed an approach used by Ruiz et al. [5] to measure “attack robustness” of the networks [19] by sequentially removing nodes from the network and measuring the size of the remaining largest connected component relative to its starting size (Fig. 2). Nodes were removed in order of decreasing betweenness (probing bottlenecks in the network) (Fig. 2a), decreasing node degree (probing hubs) (Fig. 2b), or randomly (Fig. 2c). In each case, the CDBF network was found to be more robust than the SDB and the SDF network. The attack robustness on decreasing betweenness was considerably increased in the CDBF network, consistent with the reduced modularity.

**Fig. 3:**
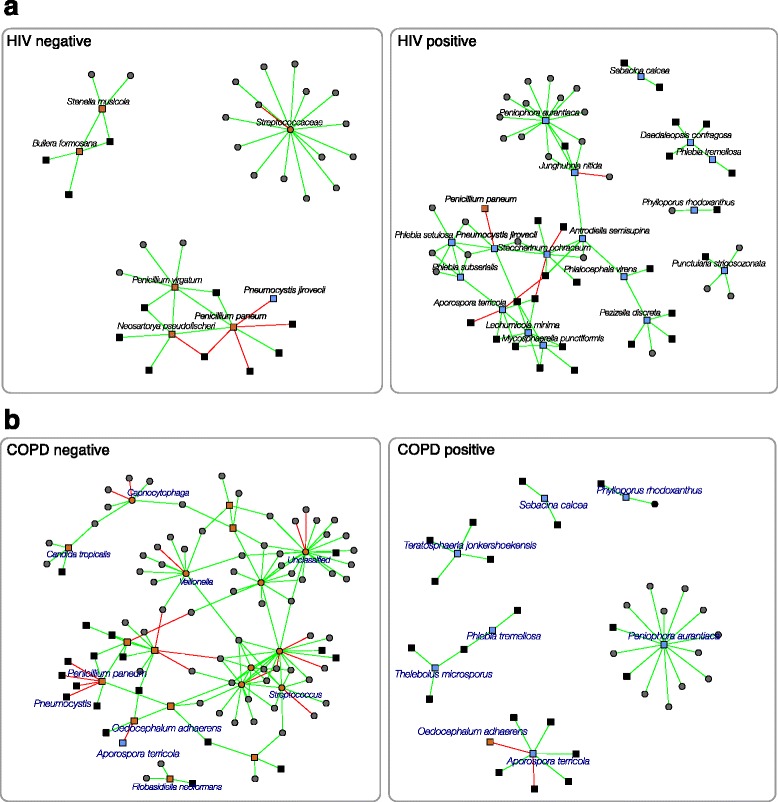
Subnetwork of exclusive OTUs with HIV+, HIV−, COPD+, and COPD− status and their nearest neighbors. Seventeen fungal OTUs were uniquely present in HIV+ individuals while one bacterial and five fungal OTUs were uniquely present in HIV− individuals. Seven fungal OTUs were uniquely present in COPD+ individuals while eight bacterial and ten fungal OTUs uniquely occurred in COPD− individuals. **a** The 17 HIV+ OTUs and their 51 nearest neighbors OTUs formed a subnetwork with five components. The HIV− subnetwork was comprised of 6 single-status nodes and 32 adjacent neighbors organized in three components. **b** The 17 COPD− OTUs with its 51 adjacent OTUs formed a large connected component with 64 members and a small four-node component. The seven COPD+ OTUs with 33 adjacent nodes organized into a disconnected six component network

**Fig. 4:**
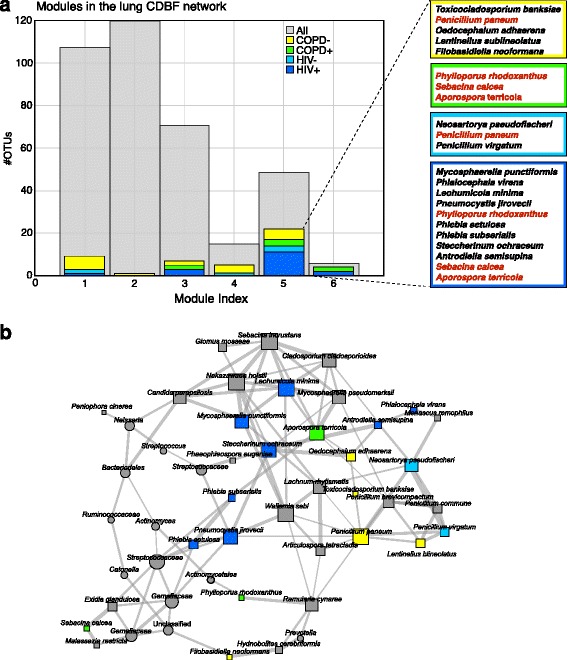
Lung microbiome modules and HIV infection/COPD status. **a** Assignment of OTUs into modules of the lung cross-domain bacterial-fungal (CDBF) network. The CDBF network is comprised of six modules. OTUs uniquely appearing in HIV-infected (dark blue), HIV-uninfected (light blue), COPD negative (yellow), or COPD positive (green) samples are found across all modules with strong enrichment in module 5. The unique species names that appeared in module 5 are listed on the right. **b** Associations in module 5 of the lung CDBF network. The size of the nodes was scaled by the number of neighbors, the thickness of the edges marks association strength

To study the relationship between HIV infection, COPD status, and the topology of the association networks, we examined OTUs uniquely present in HIV-infected (HIV+) or HIV-uninfected (HIV−) individuals, and those OTUs present only in individuals that were COPD positive (COPD+) or showed normal lung function (COPD−). The neighborhoods that comprised the status-dependent OTUs and their nearest neighbors in the lung CDBF network are shown in Fig. 3. We found that two network modules (modules 5 and 6) were enriched for OTUs that were uniquely present in any of the four status groups (Fig. 4). Module 6 included an OTU identified as *Daedaleopsis confragosa*, uniquely present in HIV+ individuals, an OTU identified as *Thelebolus microsporus* uniquely present in COPD+ individuals, and an OTU identified as *Phlebia tremellosa* present in both HIV+ and COPD+ individuals. Module 5 included 18 fungal OTUs which were uniquely present in one or more of the four status groups (Fig. 4a). The OTUs identified as *Sebacina calcea* and *Aporospora terricola* appeared uniquely in HIV+ and COPD+ individuals, whereas the OTU identified as *Penicillium paneum* appeared uniquely in HIV− and COPD− individuals. In addition to these 18 fungal OTUs, module 5 included 13 bacterial OTUs and 18 additional fungal OTUs. The network comprised of all OTUs in module 5 is shown in Fig. 4b. The gain or loss of these status-specific organisms, which may occur at the onset of COPD or HIV, has the potential to cause major restructuring of interactions within the module and across the entire network.

#### Skin microbiome

**Fig. 5:**
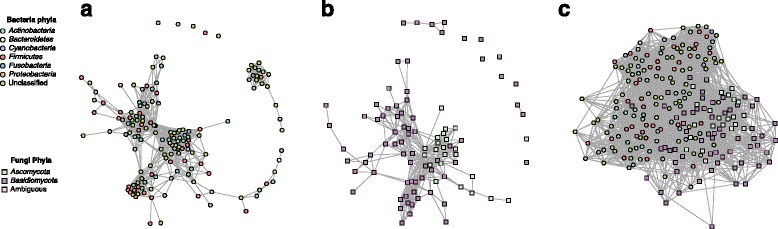
Skin microbiome networks. Networks inferred for the skin microbiome based on **a** bacteria only, **b** fungi only, and **c** bacteria and fungi combined. In all three networks, bacterial nodes are circles and fungal nodes are squares. Each node is colored by phyla. Edges between nodes represented a predicted interaction, either positive or negative

The skin SDB network was comprised of one large connected component with 130 out of 153 bacterial OTUs (84.97%), 18 OTUs (11.76%) in a small connected component, and 5 singletons (Fig. 5a). The nodes had an average degree of 11.37 (SD = 7.63). Modularity analysis of the largest component revealed four modules with realized modularity of 0.91. The skin SDB network showed highly modular organization and no central core (Estrada topology class II), and no assortativity by phylum (*c*_a_ = − 0.02).

The skin SDF network consisted of one large connected component containing 79 out of 94 fungal (84.04%) OTUs, a quintet (5.32%), a dyad (2.13%), and 8 singletons (8.51%) (Fig. 5b). The network had an average node degree of 7.51 (SD = 6.40). The main connected component was organized into five modules with realized modularity of 0.74. The SDF network belonged to Estrada class IV (mixture of core-periphery and modular network), and showed moderate phylum assortativity (*c*_a_ = 0.40).

The skin CDBF network consisted of a single connected component comprised of all 229 fungal and bacterial OTUs (Fig. 5c). The network’s average node degree was 20.02 (SD = 6.89). Modularity analysis revealed six modules with realized modularity of 0.70. The skin CDBF network belonged to Estrada class II (modular without central core) and showed low-to-moderate phylum assortativity (*c*_a_ = 0.32).

A comparison of the skin CDBF network with the single-domain networks revealed network re-organization principles similar to the lung microbial community. The average path length was shorter in the cross-domain network (mean = 2.29, SD = 0.64) than in the SDB (mean = 3.18, SD = 1.71) or SDF (mean = 2.65, SD = 1.06) networks. We observed that the ECT of the CDBF network was smaller (ECT = 562) than the SDB network (ECT = 795), implying improved global connectivity. Attack robustness analysis revealed that the cross-domain network was topographically less sensitive to species loss than the single-domain networks (Fig. 2). These features were consistent with the reduced modularity observed in the CDBF network.

### Validation of interactions in co-culture

**Fig. 6:**
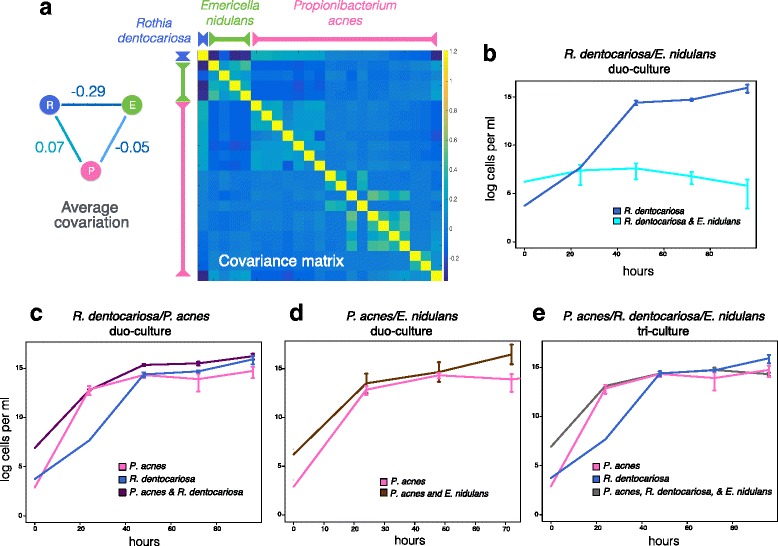
Co-variation pattern and growth curves for co-culture validation experiment. **a** *Emericella nidulans* (E, green), *Propionibacterium acnes* (P, pink), and *Rothia dentocariosa* (R, blue) form a clique in the skin CDBF network (left). The edge weights are average covariations from the estimated covariance matrix (right) between all species assigned to *R. dentocariosa* (one member), *P. acnes* (18 members), and *E. nidulans* (four members). The microbes were grown in pairs and a trio, and the growth curves for the bacteria were compared to when they were grown in monoculture. Cellular concentration growth curves are based on the average of three biological replicates and the vertical lines indicate their standard deviations. While we were able to grow *E. nidulans* as a monoculture, no growth curves are available because there is no established method for measuring the growth of filamentous fungi in liquid culture. **b** *R. dentocariosa* grown with *E. nidulans* (cyan line) or alone (blue line). **c** *R. dentocariosa* grown with *P. acnes* (purple line) or alone (blue line). **d** *P. acnes* grown with *E. nidulans* (brown line) or alone (pink line). **e** Trio of all three organisms grown together (grey line) compared to *R. dentocariosa* alone (blue line) or *P. acnes* alone (pink line)

To experimentally validate some of the cross-domain associations de novo, we established growth assays for a representative set of three species in the community. We limited ourselves to medically relevant fungi and bacteria that could be identified to the species level, could be commercially obtained, and could be grown under aerobic conditions. This last criterion eliminated all the lung microbiome cross-domain associations as all cliques that could be identified to a species level included at least one strictly anaerobic bacterium. From the skin microbiome, we selected species that formed a clique in the CDBF network, that covered a significant part of the network, and that showed non-trivial co-variation patterns across the skin samples. These criteria led us to choose the bacterium *Propionibacterium acnes*, a prominent species in the skin microbiome samples (18 OTUs were assigned to this species). We selected the fungus *Emericella nidulans* (*Aspergillus nidulans*) because four OTUs were assigned to this species that showed consistent strong negative co-variation with a third species, *Rothia dentocariosa*. The co-variation pattern of *P. acnes* with *E. nidulans* and *R. dentocariosa* was, on average, close to zero, serving as a baseline for weak overall interaction (Fig. 6a).

To measure growth, we established and compared growth curves for each bacterial species under uniform conditions, in pairs, and as a trio. The key observation of the experiment was that, as predicted, *R. dentocariosa* grew significantly worse in the presence of *E. nidulans* than when grown in monoculture (KS *p* = 0.003; Fig. 6b). For the other two pairs, no significant change in growth was detected compared to monoculture, confirming that the associations of *P. acnes* with *E. nidulans* and *R. dentocariosa* were relatively weak (Fig. 6c, d). Overall, bacterial growth appeared to be fully restored in the tri-culture at similar levels observed in *R. dentocariosa* or *P. acnes* monoculture (*p* = 0.228 and *p* = 0.925, respectively; Fig. 6e). However, the bacterial growth curve measurement technique could not discriminate *R. dentocariosa* and *P. acnes* growth, leading to two potential explanations for the observed growth. Either *P. acnes* alleviated the negative effects of *E. nidulans* on *R. dentocariosa*, resulting in joint growth of both species, or the negative effects of *E. nidulans* persisted, leading to higher abundance of *P. acnes* compared to *R. dentocariosa*. Predicting the details of this higher-level interaction involving more than two species is beyond the scope of any ecological network inference tool, including SPIEC-EASI.

### Role of Candida in cross-domain associations

**Fig. 7:**
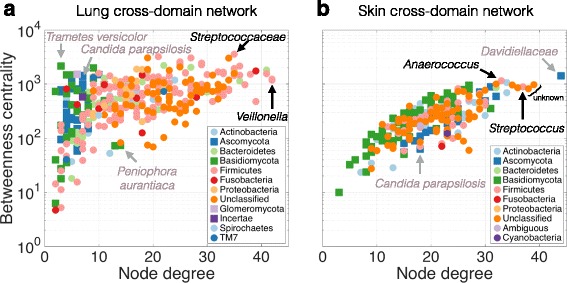
Keystone species analysis. Betweenness centrality vs. node degree of all species in the cross-domain bacterial-fungal networks of lung (**a**) and skin (**b**). Nodes with high betweenness centrality represented potential key connector (or bottleneck) species. Nodes with high degree represented hubs in the network. Both measures were indicators for potential keystone species. Bacterial species (dots) and fungal species (squares) were colored by phylum membership. Bacterial and fungal species that were maximal in either property are highlighted in both plots. One fungus in the Davidiellaceae family (top right) may act as potential keystone species in the skin microbiome. In addition, we highlighted the role of * Candida parapsilosis* across both networks

In the lung microbiome, *Candida parapsilosis*, a known fungal pathogen [20], formed associations with *Neisseria* and a member of the *Bacteroidales* order and was among the fungal species with highest betweenness centrality (Fig. 7a), suggesting a potentially important role in the lung microbial community. *C. parapsilosis* in the skin microbiome formed eight cross-domain associations with *Rothia dentocariosa*, *Propionibacterium granulosum*, *Streptococcus* spp., and five unclassified OTUs. However, *C. parapsilosis* ranked low both in terms of node degree and betweenness centrality (Fig. 7b), suggesting a less prominent role in this ecosystem. From the keystone species analysis (using betweenness centrality and node degree) (Fig. 7), we identified a fungus in the *Davidiellaceae* family as potential skin keystone species (Fig. 7b) as it maximized both measures across all species, had low relative abundance (maximum of 7.8%), and high ubiquity across samples (present in 58.1% of samples). This family contains the genera *Cladosporium* and *Davidiella*, both of which are common fungi.

## Discussion

We inferred cross-domain association networks from lung and skin microbial TAS data using our novel extension of the SPIEC-EASI framework. From lung microbial samples, we found that both the SDB and the CDBF networks had a modular organization with no central core (Estrada class II) whereas the fungal network was modular with a small central core (Estrada class IV). The inferred CDBF network showed higher overall connectivity and reduced modularity compared to both single-domain networks. Moreover, the CDBF network exhibits higher attack robustness. It has been shown that antibiotic exposure in mice resulted in lower attack robustness of the corresponding microbial association networks, compared to control [5]. This suggests that attack robustness is a promising measure for indicating destabilizing effects on microbial communities. We thus hypothesize that fungi play a stabilizing role in the microbial community organization. We also identified a network module of 49 highly connected OTUs that showed enrichment for OTUs that were uniquely present in either HIV+/− or COPD+/− individuals (18 unique OTUs). This subnetwork may see large structural rearrangements in connection with disease status and may contain key species whose presence or absence can serve as disease indicators. One candidate was the fungus *Aporospora terricola*, which was uniquely present in HIV+ and COPD+ patients and showed connections with seven other species, including *Leohumicola minima* and *Phlebia subserialis*, two plant-associated fungi uniquely identified in HIV+ patients.

The skin single- and cross-domain networks followed the same Estrada class assignment as the lung microbial networks and showed similar topological re-organization. The skin CDBF network showed increased attack robustness, more efficient global connectivity, and reduced modularity. The robustness with respect to high betweenness centrality OTUs in the network increased in the presence of fungi, suggesting an important role of fungi in the stability of the skin microbial community. From the skin CDBF network, we were able to isolate a clique containing one model fungus, *E. nidulans*, and two common bacteria, *R. dentocariosa* and *P. acnes*. By co-culturing the bacteria and fungus, we saw growth curves in line with our predicted co-variation patterns: a strong negative co-variation between *E. nidulans* and *R. dentocariosa* and near-neutral co-variation patterns between *E. nidulans* and *P. acnes*, and *R. dentocariosa* and *P. acnes*. These experiments represent, to our knowledge, the first culture-based validation of computationally predicted fungal-bacterial associations.

**Fig. 8:**
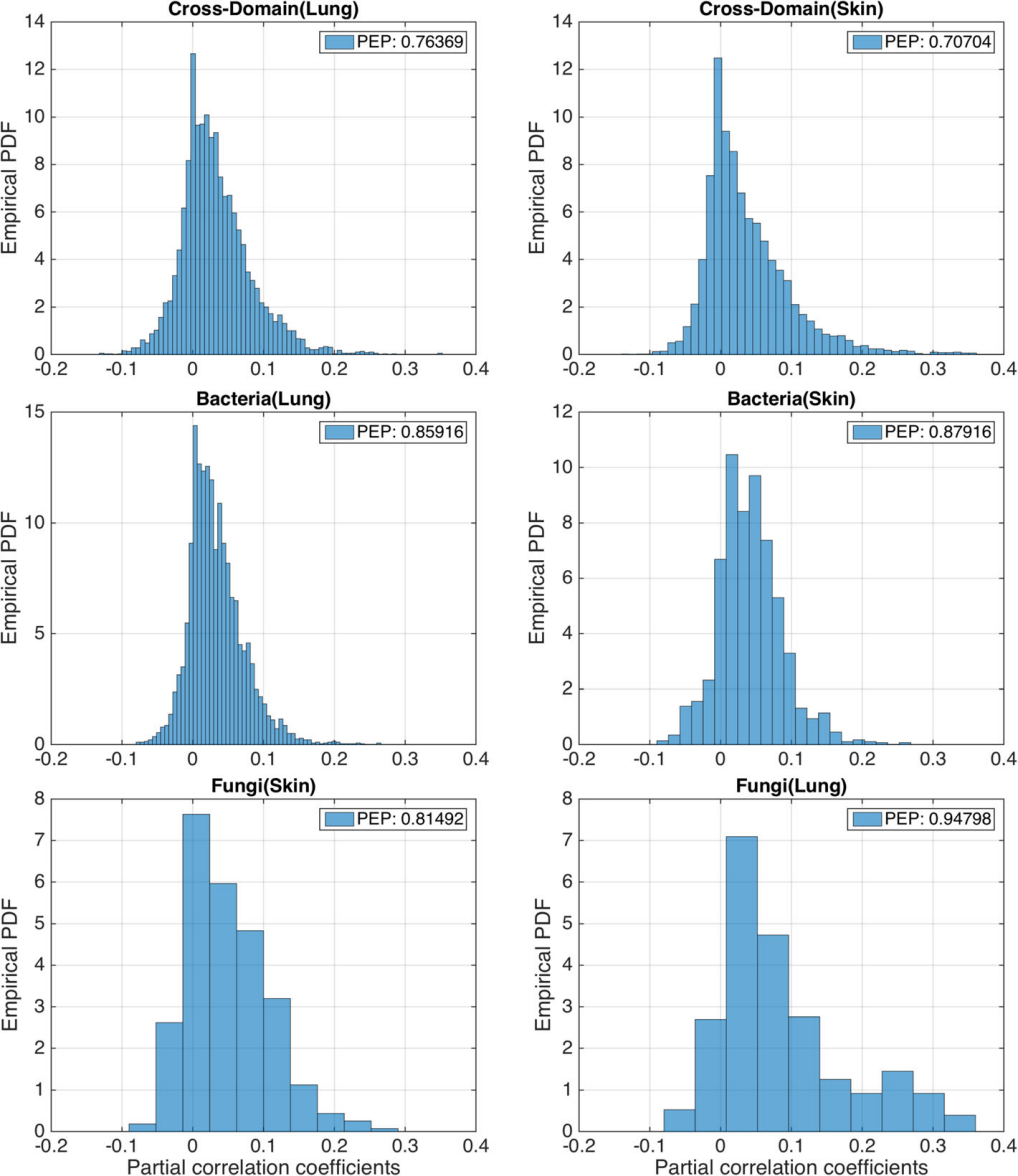
Distribution of interaction strengths (partial correlation coefficients) for all six association networks. All distributions consistently showed a peaked distribution with positive mean and skew. The positive edge percentage (PEP) was > 0.8 for all single-domain networks. Both cross-domain networks showed lower PEP (0.76 for the lung CDBF and 0.71 for the skin CDBF)

In both microbiomes, cross-domain associations reshaped the overall organization of the networks. Cross-domain interactions made up 135 out of all 2982 (4.53%) interactions in the lung microbiome and 480 out of 2292 (20.94%) interactions in the skin microbiome. The greater percentage of cross-domain interactions in the skin may have been driven by the higher biomass located there or by the greater biogeographical overlap of species from both domains on the skin. Both CDBF networks showed reduced positive-edges percentage (PEP) [21], the percentage of positive partial correlations in the networks (Fig. 8). This decrease revealed that neglecting the fungal component in the ecosystem leads to overestimation of the percentage of positive associations.

Model fungi, including *E. nidulans*, have been studied in co-culture with bacteria in the laboratory to induce properties not produced in monocultures. Direct contact with the bacterium *Streptomyces hygroscopicus* is required for *E. nidulans* to produce secondary metabolites, including polyketide synthase, often seen in nature, but not in the laboratory [22]. However, this and most other co-culture experiments originated from the knowledge that the organisms grow in physical proximity rather than from computationally predicted interactions. We have shown here that cross-domain interactions inferred computationally can be validated via co-culture in a simplified environment. Despite the simplifications, these experiments highlighted how complicated microbial interactions are likely to be and demonstrated how a tool such as SPIEC-EASI can help infer some of these interactions and provide biological insight.

## Conclusions

In summary, we have introduced a novel extension of SPIEC-EASI for predicting cross-domain associations from TAS data, applied this method to two human-associated microbiome datasets, and experimentally validated a subset of the predicted co-variation patterns. Our analysis of network features and network topologies of single- and cross-domain networks revealed that analyzing ecological association networks from a single domain may bias accurate characterization of the overall topology and robustness of the microbial ecosystem under study. Incorporating other ecosystem members, such as protists, archaea, and viruses, should be a priority for future network inference efforts. If surveyed with TAS, these taxa can be readily incorporated into the current cross-domain SPIEC-EASI framework presented here. These computational efforts, paired with large-scale culture-based association validation experiments, will help formulate concrete hypotheses about the underlying biological mechanisms of species interactions and, ultimately, help understand microbial ecosystems as a whole.

## Methods

### Adapting SPIEC-EASI for two domains

We adapted the SPIEC-EASI method to analyze microbiome networks across multiple microbial domains [2]. The tables of absolute bacteria and eukaryote OTU counts are stored in matrices = $W\in {\mathrm{\mathbb{N}}}_0^{n\times d},\kern0.5em V\kern0.5em \in {\mathrm{\mathbb{N}}}_0^{n\times p}$, where ${w}^{(j)}=\left\{{w}_1^{(j)},{w}_2^{(j)},\dots, {w}_d^{(j)}\right\}$ and${\upsilon}^{(j)}=\left\{{\upsilon}_1^{(j)},{\upsilon}_2^{(j)},\dots, {\upsilon}_p^{(j)}\right\}$ denote the *d*- and *p*-dimensional row vectors of counts from the *j*th sample, with *j* ∈ {1*,...,n*} and *N*_0_ denotes the set of natural numbers. Define the total cumulative counts for each domain as *M*^*(*^^*j)*^ = $\sum \limits_{i=1}^d{w}_i^{(j)}$ and *N*^(^^*j*^^)^ = $\sum \limits_{i=1}^p{v}_i^{(j)}$.

In a standard sequencing experiment, absolute count data *w*^(*j*)^ and *v*^(*j*)^ are unknown, since absolute information is typically not available. However, by dividing observed sequencing counts by the total library size, we get compositional data vectors, ${x}^{(j)}=\left\{{x}_1^{(j)},{x}_2^{(j)},\dots, {x}_d^{(j)}\right\}$ and ${y}^{(j)}=\left\{{y}_1^{(j)},{y}_2^{(j)},\dots, {y}_p^{(j)}\right\}$, with associated relative abundance matrices $X\in {\mathbb{S}}^{d\times n}$ and $Y\kern0.5em \in \kern0.5em {\mathbb{S}}^{p\times n}.\kern1em {\mathbb{S}}^p\doteq \left\{x\left|{x}_i\right.>0,{\sum}_{i=1}^p{x}_i=1\right\}$ is the *p*-dimensional unit simplex. It is well known that components of a composition are not independent due to the unit sum constraint, and covariance matrices of compositional data show negative bias due to closure. Furthermore, compositional data can be completely determined from absolute abundance data (termed a basis), i.e.,${x}^{(j)}=\left\{{w}_1^{(j)}/{M}^{(j)},{w}_2^{(j)}/{M}^{(j)},\dots, {w}_d^{(j)}/{M}^{(j)}\right\}$.

As noted by John Aitchison, the simple equivalence

1 $$ \log \left[\frac{x_i}{x_j}\right]=\log \left[\frac{w_i/M}{w_j/M}\right]=\log \left[\frac{w_i}{w_j}\right], $$

implies that statistical inferences drawn from the analysis of log-ratios of compositions are equivalent to those drawn from analysis of log-ratios of the basic components, which establishes the precedence of log-ratio transformations to study compositional data. The centered log-ratio (CLR) transformation,

CLR(*x*) = $\left\{\log \left[\frac{x_1}{g(x)}\right],.\kern0.5em .\kern0.5em .\kern0.5em ,\log \left[\frac{x_d}{g(x)}\right]\right\}$, where $g(x)={\left[\prod \limits_{i=1}^d,{x}_i\right]}^{1/d}$, is particularly useful, as it is symmetric and isometric with respect to the original composition. The CLR maps compositional data from the simplex to a (*d* − 1)-hyperplane of *d*-dimensional Euclidean space, with the corresponding population covariance matrix Γ_*X*_ = Cov[CLR(X)]. The matrix Γ_*X*_ is related to the population covariance of the log-transformed absolute abundances Ω_*W*_ = Cov[log*W*] by

2 $$ {\Gamma}_x={G}^d{\Omega}_W{G}^d, $$

where ${G}^d={I}^d-\frac{1}{d}{\mathbf{11}}^T$, is the standard centering matrix, where *I*^*d*^ is the *d* × *d* identity matrix and **1** = {1, …, 1}, the *d*-length vector of ones. Therefore, for high-dimensional data, *d* ≫ 4 ,

3 $$ {G}^d\approx {I}^d $$

and Γ_*X*_ ≈ Ω_*W*_ is a reasonable approximation. Indeed, recent work has shown the theoretical conditions under which the covariance structure Ω_*W*_ is approximately identifiable from Γ_*X*_, where recovery guarantees depend on sparsity, dimensionality, and sample size [23].

This observation was the basis of SPIEC-EASI, which seeks to estimate a sparse inverse covariance (precision) matrix using the sample covariance as input to the optimization problem

4 $$ {\widehat{\Omega}}_W^{-1}={\mathrm{argmin}}_{{\widehat{\Omega}}_W^{-1}\in PD}-\log \det \left({\widehat{\Omega}}_W^{-1}\right)+\mathrm{tr}\left({\widehat{\Omega}}_W^{-1}{\widehat{\Gamma}}_x\right)+\lambda \parallel {\widehat{\Omega}}_W^{-1}{\parallel}_1 $$

where ${\widehat{\varGamma}}_x$ is the sample covariance estimate of CLR(*X*) and *PD* is the set of symmetric positive definite matrices. Solving Eq. 4 ensures that the penalized estimator is full rank. The sparsity pattern depends on the value of *λ*, since the L1 norm ||.||_1_ penalizes the absolute values of the entries of the symmetric precision matrix. The collection of non-zero entries of ${\widehat{\varOmega}}_W^{-1}$ is then interpreted as the graph associated with the single-domain microbial community under study*.*

In cross-domain studies, different marker genes are amplified and sequenced separately and, hence, do not compete for reads. This implies that technically independent compositions are generated in these studies. Therefore, a naive application of Eq. (4) directly to the combined dataset [ *X Y* ], a *n* × (*d* + *p*) matrix generated from a simple concatenation of two compositional datasets, would be inappropriate.

To illustrate this, consider that the log-ratio

5 $$ \log \left[\frac{x_i}{y_j}\right]=\log \left[\frac{w_i/M}{\upsilon_j/N}\right]\ne \log \left[\frac{w_i}{\upsilon_j}\right]\kern1em $$

does not satisfy the scale-invariance property of Eq. 1. Similarly, the approximation in Eq. (3) does not hold between cross-compositional pairs.

We instead considered the data matrix *Z* = [ CLR(*X*) CLR(*Y*) ], generated by concatenating independently transformed compositions. Γ_*Z*_ = Cov[*Z*] has the still-satisfying relation to the basis covariances:

6 $$ {\Gamma}_Z=\left[\begin{array}{l}\kern0.5em {G}^d{\Omega}_W{G}^d\kern1em {G}^d{\Omega}_{WV}{G}^p\\ {}{G}^p{\Omega}_{VW}{G}^d\kern2em {G}^p{\Omega}_V{G}^p\kern0.5em \end{array}\right], $$

where ${\mathsf{\varOmega}}_{\mathit{\mathsf{W}\mathsf{V}}}=\mathsf{Cov}\left[\mathsf{\log}\mathit{\mathsf{W}},\mathsf{\log}\mathit{\mathsf{V}}\ \right]$ is the cross-covariance matrix between the two log-transformed basis datasets, and ${\mathsf{\varOmega}}_{\mathit{\mathsf{VW}}}={\left({\mathsf{\varOmega}}_{\mathit{\mathsf{WV}}}\right)}^{\mathit{\mathsf{T}}}$. In other words, the $\left(\mathit{\mathsf{d}}+\mathit{\mathsf{p}}\right)\times \left(\mathit{\mathsf{d}}+\mathit{\mathsf{p}}\right)$ combined covariance structure Γ_*Z*_ is decomposable into blocks where the approximation in Eq. (3) holds. If *p*,*d* >>4 then the approximation

7 $$ {\Gamma}_Z\approx {\Omega}_z=\left[\begin{array}{l}{\Omega}_W\kern1.5em {\Omega}_{WV}\\ {}{\Omega}_{VW}\kern2em {\Omega}_V\end{array}\right] $$

allows us to use ${\widehat{\varGamma}}_Z$ as the input to Eq. (4) to get a penalized estimator ${\widehat{\varOmega}}_Z^{-1}$, which is interpretable as an intra- and cross-domain interaction network and amenable to the standard SPIEC-EASI framework. The same principle can be applied to more than two domains.

### Datasets

In this study, we analyzed two previously published microbiota datasets that included both bacterial and fungal sequences. The first was from bronchoalveolar lavages (BALs) collected as part of the Lung HIV Microbiome Project, as published in [10] and [11]. It contained 35 samples that were subjected to 16S rRNA gene and ITS sequencing. The BAL samples originated from the right middle lobe or the left upper lobe of the lungs from 25 individuals of whom 14 were HIV-infected and 11 were HIV-uninfected. Of the 35 samples, 17 came from individuals with normal spirometry and 18 from individuals with COPD (diffusing capacity of the lungs from carbon monoxide (DLCO) < 80% or forced expiratory volume in 1 s (FEV1) < 70%). The demographics of the cohort analyzed here can be found in Table 1. No significant differences were found between HIV-infected and HIV-uninfected or between individuals with COPD and those with normal lung function.

**Table 1:** Demographics of the lung microbiome cohort. Values presented as mean (SD) except for those that are the percentage of the subset denoted with (%). *P* values are from Welch *t* tests for continuous variables and from Fisher’s exact tests for percentages

	Cohort	HIV+	HIV−	*p*-value	COPD+	COPD−	*p* value
*N*	25	14	11	–	13	12	–
Age (years)	51.5 (7.7)	51.2 (8.3)	51.9 (7.4)	0.8472	49.4 (8.1)	53.6 (7.1)	0.2032
Male (%)	88.0	92.9	81.8	0.5648	92.3	83.3	0.5930
White (%)	56.0	50.0	63.6	0.6887	53.8	58.3	1.0000
Current smokers (%)	20.0	28.6	9.1	0.4913*	30.8	8.3	0.4671*
Former smokers (%)	12.0	14.3	9.1		7.7	16.7	
BMI (kg/m^2^)	25.9 (5.3)	24.2 (4.2)	28.1 (5.9)	0.0792	24.4 (5.4)	27.6 (4.8)	0.1426
Viral load (IU/mL)	–	1477 (2850)	–	–	2053.5 (3230.5; *N* = 10)	35.5 (18.2; *N* = 4)	0.0746
CD4 count (cells/mm^3^)	–	646 (305)	–	–	620.2 (326.2; *N* = 10)	701.8 (278.8; *N* = 4)	0.6195
FEV1/FVC (%)	79.0 (11.5)	80.1 (8.8)	77.7 (14.6)	0.6435	75.8 (15.0)	82.5 (4.1)	0.1463
DLCO (ml/min/mmHg)	77.2 (15.3)	73.3 (16.0)	82.1 (13.5)	0.1520	66.8 (13.9)	88.4 (6.0)	< 0.0001

^*^Smoking status *p* value calculated using an ANOVA test

The second dataset was from a skin microbiome study at the National Human Genome Research Institute, as published in [12] and [13]. It includes 382 samples from 14 body sites on 10 healthy adults. Ten body sites were repeated on the left and right sides, and some of the healthy volunteers underwent repeat sampling 1–3 months after their initial visits.

### Sample and sequence processing

Sample processing procedures for the lung microbiome have been previously described [10, 11]. In brief, all samples had DNA extracted using standard techniques of the PowerSoil® DNA Isolation Kit from MO BIO (Carlsbad, CA). For bacterial DNA sequencing, the hyper-variable regions 1 through 3 (V1–V3) of the 16S rRNA gene were amplified and sequenced using the Roche 454 GS-FLX Titanium platform. For fungal DNA sequencing, the ITS1 was amplified and sequenced on the Ion PGM™ Sequencer using the 400 bp protocol [24].

The sample processing procedures for the skin microbiome were previously described [12, 13]. In brief, samples were lysed using the MasterPure™ Yeast DNA Purification Kit, cell walls were mechanically disrupted using a Tissuelyser (Qiagen, Valencia, CA), and DNA was extracted using the Invitrogen PureLink Genomic DNA Kit (Invitrogen, Carlsbad, CA). For bacteria DNA sequencing, the V1–V3 regions of the 16S rRNA gene were amplified, and for fungal DNA sequencing, the ITS1 region was amplified. Both bacterial and fungal DNA was sequenced on the Roche 454 GS20/FLX platform with Titanium chemistry (Roche, Branford, CT). We analyzed the resulting sequences in a manner consistent with the lung microbiome, which was different than that which was used in the original publications.

All sequences from both the lung and skin microbiomes were processed using the QIIME pipeline version 1.7 [25] with default settings for the de novo Operational Taxonomic Unit (OTU) picking at 97% similarity for bacteria and 99% similarity for fungi. Additional processing for the ITS sequences was performed using FHiTINGS [26]. Samples with fewer than 1000 16S bacterial reads (*N* = 16 for the lung microbiome; *N* = 12 for the skin microbiome) and samples with fewer than 50 ITS fungal reads (*N* = 16 for the lung mycobiome; *N* = 3 for the skin mycobiome) were considered to have failed and were removed. Bacterial taxonomic assignments were made using the Green genes 12.10 reference database [27], and fungal taxonomic assignments were made using the FHiTINGS version of the Index Fungorum (http://www.indexfungorum.org/) reference database [26].

We removed OTUs present in fewer than 1/3 of the samples (20 lung samples or 120 skin samples) as well as any OTUs represented by single reads in every sample. Requiring that OTUs be present in 1/3 or more of the samples reduces the influence of any single individual, since both environments included repeated samples of the same individual volunteer. The number of samples and bacterial and fungal OTUs of each resulting network dataset are presented in Table 2. A pseudo count of 1 read was added to every OTU in every sample to eliminate zeros in samples where OTUs were absent. All OTU counts were normalized using total sum scaling (also known as relative abundance) followed by centered log-ratio scaling [28], as described above.

**Table 2:** Dataset sizes for each network constructed. Amplification of target genes and sequencing were not successful for all samples resulting in variable node counts in the combined networks

Network	Samples	Bacteria OTU nodes	Fungi OTU nodes
Lung bacteria only	77	302	–
Lung fungi only	48	–	96
Lung combined	35	302	68
Skin bacteria only	360	153	–
Skin fungi only	375	–	94
Skin combined	353	144	85

### Constructing networks

All networks were constructed using the *SpiecEasi* package version 0.1 in R (https://github.com/zdk123/SpiecEasi). We used the sparse graphical lasso (glasso) setting and selected the optimal sparsity parameter based on the Stability Approach to Regularization Selection (StARS) [29]. The StARS variability threshold was set to 0.1 for all networks.

### Evaluating and comparing networks

Networks were analyzed using functions of the R package *igraph* version 1.0.1 [30] and customized MATLAB scripts. We evaluated node degree (i.e., the count of edges a node has) as a measure of sparsity. A complete network would have an average node degree equal to the number of nodes minus 1; a lower degree indicates a sparser network. To evaluate connectedness of the networks, we used normalized node betweenness centrality for undirected graphs. Normalized node betweenness centrality measures the proportion of the shortest paths in the network that pass through the node. A lower average betweenness centrality number indicates a more connected network, either because of more shortest paths or because fewer of the shortest paths travel through each node. These metrics, as well as distance between nodes, were used to compare the networks using Welch’s unequal variances t-tests [31]. We used expected commute time (ECT) as a measure of connectedness [17]. For each pair of nodes, the commute time corresponds to the expected number of “hops” it takes for a random walk on the graph to visit the paired node and to return to the starting node. The expected commute time is an average over all pairs of nodes and can be efficiently computed for the spectrum of the graph [17]. Modularity analysis was performed using the standard deterministic modularity maximization framework [14]. The reported realized modularity measure, introduced in [15], Eq. 2, is the ratio of the number of edges within modules and the number of edges across modules. Following Estrada [16], we determined the topological (or Estrada) class of the different networks using the concept of subgraph centrality and spectral scaling (see [16] for details). Briefly, Estrada introduced four topological classes for complex networks. Networks in class I possessed good expansion properties, implying that the network cannot be partitioned into separate modules. Class II was comprised of networks with modular structures with smaller interconnectivity among modules. Class III was comprised of networks with core-periphery structure, implying a tightly connected core module with sparse branches to nodes in the periphery. Class IV networks possessed features of both class II and class III networks. MATLAB analysis scripts are available at https://github.com/muellsen/MNA/tree/master/Tipton-2017.

### Microbial co-cultures

All organisms were purchased from ATCC and grown under their recommended conditions (Table 3) to establish stocks. From these stocks, uniform condition stocks were inoculated in 10 mL brain-heart infusion (BHI) broth in 74-mm^2^-untreated Nest Biotechnology (Wuxi, China) culture flasks and incubated at 37 °C under aerobic conditions with mild shaking. BHI was selected as the growth medium because all three organisms have been shown to grow on it. The established uniform conditions were used to grow mono, dual, and tri organism co-cultures, each started with the same number of cells of each organism (10 million bacterial cells or 1 million fungal spores). Growth was measured by cellular concentration, calculated based on cell counts in five locations on a hemocytometer, every 24 h for 5 days, and curves were fit by connecting the average of three biological replicates. To ensure that cells were maintaining viability, aliquots were plated on BHI agar at each time point and colony forming units (CFUs) were counted after a 24-h incubation period.

**Table 3:** Organisms and their recommended growing conditions. Each of the three microbes used in co-culture validation experiments was purchased from ATCC, rehydrated, and grown under their recommended conditions before co-culturing began. The ATCC catalogue numbers and recommended growing conditions are presented here. Because *P. acnes* is an aerotolerant anaerobe, it was first grown in a homemade anaerobic jar inside the incubator

Organism	ATCC catalogue number	Recommended temperature	Recommended media
*Emericella nidulans*	96,921	24 °C	Malt extract agar
*Propionibacterium acnes*	6919	37 °C	Tryptic soy agar with 5% sheep blood
*Rothia dentocariosa*	17,931	37 °C	Brain-heart infusion agar

## Abbreviations

16S rRNA gene: 16S subunit of the ribosomal RNA gene
BAL: Bronchoalveolar lavage
BHI: Brain-heart infusion
CDBF: Cross-domain bacterial-fungal (network)
CFUs: Colony forming units
CLR: Centered log-ratio
COPD: Chronic obstructive pulmonary disease
DLCO: Diffusing capacity of the lungs from carbon monoxide
ECT: Expected commute time
FEV1: Forced expiratory volume in 1 s
HIV: Human immunodeficiency virus
ITS: Internal transcribed spacer
OTU: Operational taxonomic unit
PEP: Positive-edge percentage
SDB: Single-domain bacterial (network)
SDF: Single-domain fungal (network)
SPIEC-EASI: Sparse InversE Covariance estimation for Ecological ASociation Inference, pronounced “speak easy”
StARS: Stability approach to regularization selection
TAS: Targeted amplicon sequencing
